# Improvement of Culture Conditions and Plant Growth Regulators for In Vitro Callus Induction and Plant Regeneration in *Paeonia lactiflora* Pall.

**DOI:** 10.3390/plants12233968

**Published:** 2023-11-25

**Authors:** Wenhui Song, Yaohong Song, Xueting Liu, Xiaoju Zhang, Rujie Xin, Siyang Duan, Shixin Guan, Xiaomei Sun

**Affiliations:** 1 College of Forestry, Shenyang Agricultural University, Shenyang 110866, China; 2023220688@stu.syau.edu.cn (W.S.); songyaohongh@126.com (Y.S.); liuxueting@419.com.cn (X.L.); xiaojuzhang@126.com (X.Z.); xinrujie2022@stu.syau.edu.cn (R.X.); duansiyang1994@126.com (S.D.); 2 Key Laboratory of Forest Tree Genetics Breeding and Cultivation of Liaoning Province, College of Forestry, Shenyang Agricultural University, Shenyang 110866, China

**Keywords:** callus, organogenesis, shoot bud differentiation, rooting, *Paeonia lactiflora*

## Abstract

Owing to its high ornamental, medicinal and horticultural values, herbaceous peony (*Paeonia lactiflora* Pall.) has been widely used as a landscaping and economical plant around the world. However, the lack of an efficient and stable regeneration system in *P. lactiflora* restricts its rapid propagation and large-scale production. By testing the key factors affecting callus formation, proliferation, adventitious bud induction and rooting, here, we developed an in vitro system for callus induction and regeneration in *P. lactiflora*. Our results show that callus formation was affected by explant types, culture environment, basal medium and plant growth regulators. Using cotyledons as explants, we established good conditions for *P. lactiflora* callus induction and callus proliferation. We effectively obtained adventitious buds differentiated from callus in Murashige and Skoog (MS) medium containing kinetin (KT) and thidiazuron (TDZ). Adventitious bud growth can be further promoted by adding gibberellin 3 (GA_3_), 1-naphthaleneacetic acid (NAA) and 6-benzyleaminopurine (6-BA) into the MS medium. A high percentage of rooting can be achieved by adding indolebutyric acid (IBA) and activated carbon (AC) to ½ MS medium. Overall, our system promotes callus induction and adventitious bud regeneration for *P. lactiflora* through improved culture conditions and plant growth regulators in the culture media, and lays a foundation for subsequent genetic engineering research.

## 1. Introduction

Herbaceous peony (*Paeonia lactiflora* Pall.) is documented as the oldest ornamental perennial herbaceous flower in the family Paeoniaceae. Due to its graceful appearance, rich color, broad application value, and various application forms, *P. lactiflora* has fascinated people over the years, and it has also enjoyed the reputation of “Mayflower God” [1,2,3]. Studies have also shown that *P. lactiflora* roots have important medicinal values, with their therapeutical effects on cardiovascular and immune system diseases [4,5,6].

Seed reproduction has long been the major mode of propagation of *P. lactiflora* [7]. The traditional reproduction method has a long cycle with a low reproduction coefficient, and seed germination is strongly dependent on the season. After sowing for 4–5 years, well-grown plants can normally flower, but they cannot maintain the intact characteristics of the female reproductive organs [8,9,10]. Conventional growing methods are not conducive to breeding and cultivar improvement, thus tissue culturing has become an effective means for rapid/mass propagation and germplasm conservation in *P. lactiflora* [11]. Tissue culturing can also mitigate seed hypoplasia, germination difficulties and genetic instability in hybrid progeny of *P. lactiflora* [12]. Therefore, propagation using tissue culture technology provides a favorable technical approach for the large-scale regeneration and production in *P. lactiflora*.

Plants can be regenerated in vitro through direct and indirect methods. The establishment of an indirect regeneration system is one of the key prerequisites for the development of *Agrobacterium*-mediated genetic transformation and genome editing [13]. The indirect shoot regeneration scheme based on callus, or indirect somatic organogenesis, can be divided into three basic stages—callus induction, callus proliferation, and callus regeneration into shoots followed by multiplication [14].

In *P. lactiflora*, different works have been published that explore explant selection, callus formation, proliferation and shoot regeneration [15,16,17,18,19,20,21,22,23,24,25,26]. Thus, various explants have been used for callus regeneration, including stem segments [16,17,18], leaves [9,19], roots [20], cotyledons [21,22] and various floral organs [11,23]. In the callus induction stage, the explants are transferred to the Murashige and Skoog (MS) [27] medium [22,23,24] supplemented with 2,4-Dichlorophenoxyacetic acid (2,4-D), 1-naphthaleneacetic acid (NAA), thidiazuron (TDZ), kinetin (KT) or 6-benzyleaminopurine (6-BA) [22,23,24] to dedifferentiate the explants into callus. During shoot induction, the callus is transferred to the medium rich in plant growth regulators to form new adventitious shoots, and cytokinin plays an important role in shoot induction [24]. Previous studies have shown that callus and a small amount of shoots can be successfully induced with TDZ [24,25], but the subsequent rooting of shoots has not been reported [19,24,25,26]. The induction of embryogenic callus and a subsequent somatic embryo in *P. lactiflora* is hard to achieve, with only a few reports in the literature [21,23]. At the same time, there are still many problems in the process of callus formation, including low callus formation in explants, difficulty in inducing embryogenic callus, serious browning in the process of induction and proliferation, and difficulty in achieving redifferentiation, all of which seriously restricts the establishment of the regeneration system in *P. lactiflora* [11,12,19,22,23]. If the above problems can be solved, the propagation coefficient and regeneration frequency of *P. lactiflora* will be substantially improved, and an efficient and stable regeneration system of *P. lactiflora* can be built. Subsequently, the factory production of *P. lactiflora* could be established, and the utilization and development of *P. lactiflora* could be carried out in combination with various cultivation purposes to produce positive social and economic benefits, and to provide a reference for *P. lactiflora* breeding, variety improvement, and genetic transformation [28,29,30]. In this study, we improved the culture conditions and the plant growth regulators in the culture media for callus induction and regeneration in *P. lactiflora*. Our results lay a foundation for subsequent genetic engineering research.

## 2. Results

### 2.1. Effects of Explant Types and Auxins Combined with 6-BA on Callus Induction

**Figure 1:**
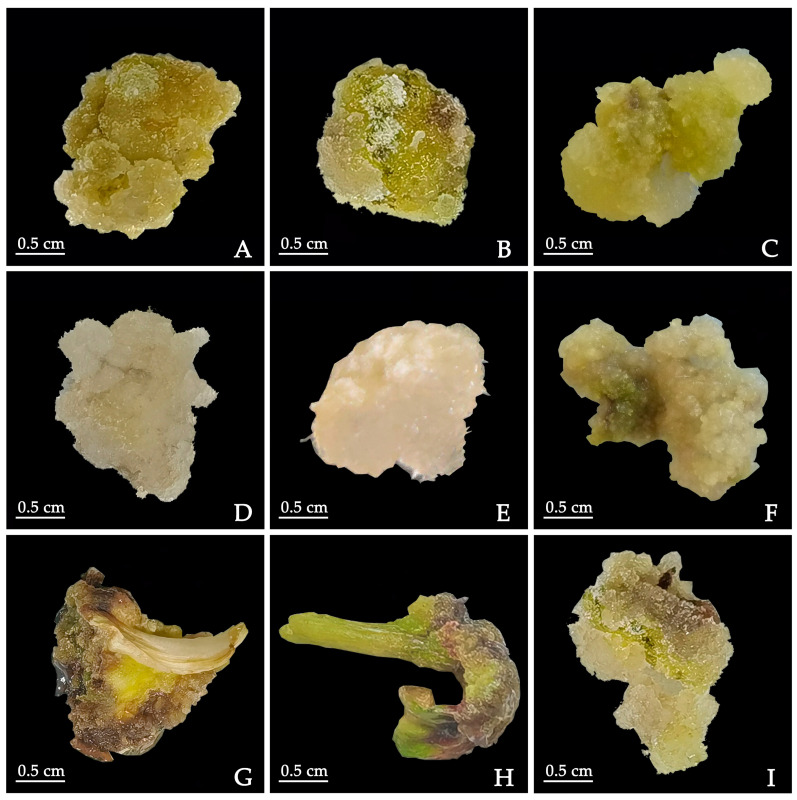
Induction of callus of *P. lactiflora* with explant types and auxins combined with 6-BA. (**A**–**C**) Calluses induced from cotyledon, hypocotyl and embryo in 1.0 mg·L^−1^ NAA, respectively. (**D**–**F**) Calluses induced from cotyledon, hypocotyl and embryo in 2.0 mg·L^−1^ 2,4-D, respectively. (**G**–**I**) Calluses induced from cotyledon, hypocotyl and embryo in 2.0 mg·L^−1^ PIC, respectively. These results were obtained after 45 days of cultivation. 6-BA: 6-benzyleaminopurine; PIC: picloram; 2,4-D: 2,4-Dichlorophenoxyacetic acid; NAA: 1-naphthaleneacetic acid.

Three kinds of explants, including cotyledon, hypocotyl, and embryo, were used, and all formed regular and healthy callus in MS medium supplemented with 1.0 mg·L^−1^ 6-BA and either 1.0 mg·L^−1^ NAA or 2.0 mg·L^−1^ 2,4-Dichlorophenoxyacetic acid (2,4-D). The callus induction rate ranged between 83.89% and 98.89% (Table 1). However, the callus induction rate from cotyledon explants in the three auxins, which can be as high as 98.89%, was better than that observed in hypocotyl and embryo explants (Table 1). Morphologically, the callus induced from cotyledon and hypocotyl explants in 1.0 mg·L^−1^ NAA was compact and light yellow, and the growth was strong (Figure 1A,B). The callus of the embryo in the medium supplemented with 1.0 mg·L^−1^ NAA expanded at the base of the embryo, and it contained a compact callus with a light yellow, transparent and watery appearance (Figure 1C). Furthermore, the surface of the cotyledon-derived callus and the hypocotyl-derived callus in 2.0 mg·L^−1^ 2,4-D was white-flocculent, soft, and fragile (Figure 1D,E). The callus of embryo in the medium supplemented with 2.0 mg·L^−1^ 2,4-D was compact, light yellow and transparent (Figure 1F). At 2.0 mg·L^−1^ PIC, the callus formed by cotyledon and hypocotyl was poorly dedifferentiated with a hard texture and obvious brown surface (Figure 1G,H). The callus induction rate from embryo explants was low in the medium supplemented with 2.0 mg·L^−1^ PIC, and the site of occurrence was not fixed (Figure 1I). Overall, the appearances of the callus from the three explants were quite different.

### 2.2. Effects of Basal Medium and NAA Concentration on Callus Induction

There were differences in callus induction efficiency and growth status between the two basal media when cotyledons were used as the explants. Our experiments show that the browning rate of MS medium was generally lower than that of ½ MS medium with the same concentration of NAA, and the induction speed and callus status of MS medium were also better than that of ½ MS medium (Table 2). Especially, when MS was used as the basal medium, we observed the best callus induction rate (98.33%) and lowest browning rate (1.67%) when 0.5 mg·L^−1^ TDZ + 0.5 mg·L^−1^ 2,4-D + 0.5 mg·L^−1^ NAA was added. Morphologically, the callus, which was conducive to further proliferation and differentiation, was yellowish-green, compact, and moderately hard. When ½ MS medium was used, the optimal combination was 0.5 mg·L^−1^ TDZ + 0.5 mg·L^−1^ 2,4-D + 1.0 mg·L^−1^ NAA. In summary, the optimal medium for callus induction was MS + 0.5 mg·L^−1^ TDZ + 0.5 mg·L^−1^ 2,4-D + 0.5 mg·L^−1^ NAA.

### 2.3. Effects of Dark Culture Time on Callus Induction

**Figure 2:**
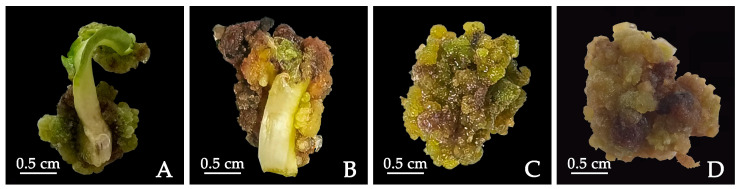
Growth states of calluses from cotyledon of *P. lactiflora* under different dark culturing times. (**A**) Calluses were incubated in the dark for 0 days. (**B**) Calluses were incubated in the dark for 10 days. (**C**) Calluses were incubated in the dark for 20 days. (**D**) Calluses were incubated in the dark for 30 days. After dark culturing, they were transferred to light culture conditions. After 30 days of cultivation, the images were collected.

Though there was no significant difference in callus induction rate under different dark treatments, the callus browning rate was significantly different (Table 3). Calluses that had not undergone dark treatment or had undergone short dark culture times had hard textures and the cotyledon was incompletely dedifferentiated (Figure 2A,B). The calluses from cotyledon explants cultured in the dark for 20 days had the best condition with a low browning rate and light yellowish color, and part of the callus surface was convex and granular. As the cultures were moved into the light, the calluses gradually changed from light yellow to yellowish-green, and the growth status was improved (Figure 2C). Calluses cultured in the dark for a longer time (e.g., 30 days) were observed to have a black-yellow color, friable/watery surface, loosely held surface cells, and obvious browning/poor state (Figure 2D). In summary, culturing under dark conditions can affect the growth states of calluses. We demonstrated that 20 days of dark culturing was the best time in terms of inducing calluses using cotyledon as an explant.

### 2.4. Effects of PIC on Callus Induction

**Figure 3:**
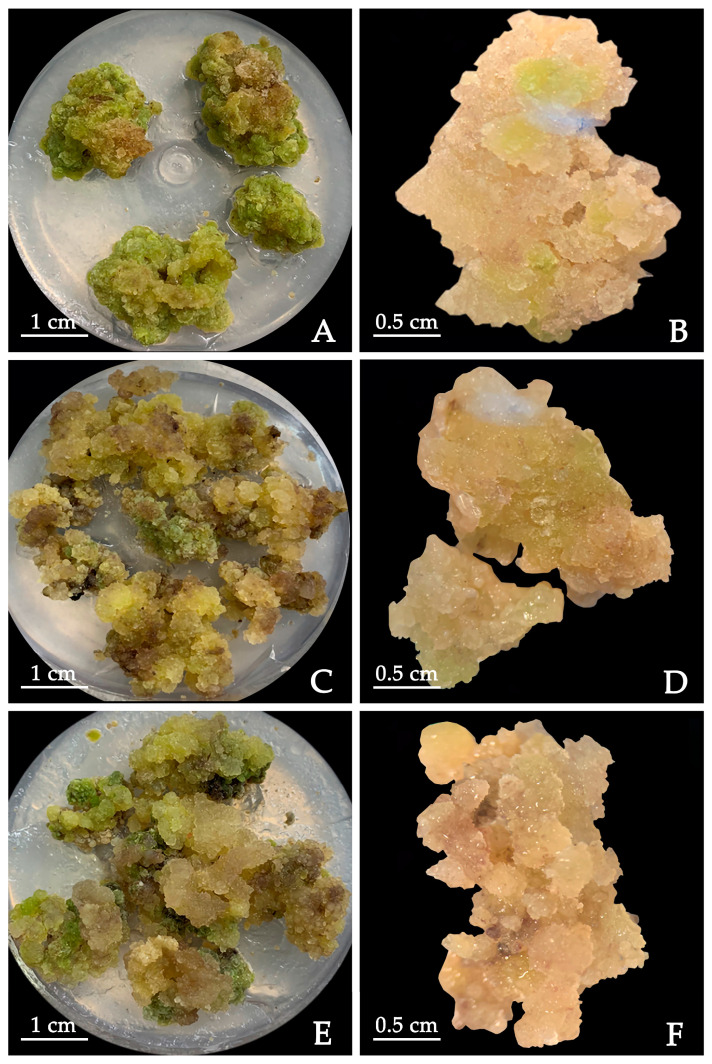
Induction of calluses from cotyledon of *P. lactiflora* with different PIC concentrations: (**A**) 0 mg·L^−1^ PIC, (**B**) 0 mg·L^−1^ PIC under stereomicroscope, (**C**) 2.0 mg·L^−1^ PIC, (**D**) 2.0 mg·L^−1^ PIC under stereomicroscope, (**E**) 4.0 mg·L^−1^ PIC, (**F**) 4.0 mg·L^−1^ PIC under stereomicroscope. PIC: picloram.

PIC has been used as a plant growth regulator in tissue culturing. Thus, we tested how PIC and its concentration can impact the callus induction of *P. lactiflora* in ½ MS (Ca^2+^) + 1.0 mg·L^−1^ TDZ + 0.5 g·L^−1^ Casein hydrolyzed (CH) + 1.0 mg·L^−1^ PVP when the proliferative callus obtained from cotyledon was used as the material. Our experiments show that the induction rate of calluses was positively correlated with the concentration of PIC. For example, the induction rate of calluses at 2.0 mg·L^−1^ PIC (46.67%) was significantly higher than that at 0 mg·L^−1^ PIC (20.00%) (Table 4). In 0 mg·L^−1^ PIC medium, the callus surface was compact and yellowish-green (Figure 3A,B). The surfaces of calluses in 2.0 mg·L^−1^ PIC medium looked healthier with a light yellowish color and looseness. We observed obvious yellow convex particles under the stereomicroscope, though some calluses showed browning (Figure 3C,D). However, when the PIC concentration increased to 4.0 mg·L^−1^, the calluses internally showed more serious browning, and the browning rate was much higher (46.67%) (Figure 3E,F). The conclusion is that PIC promotes the formation of calluses, but the appropriate concentration has yet to be established. In the case of *P. lactiflora* tissue culturing, the optimal concentration of PIC would be 2.0 mg·L^−1^.

### 2.5. Effects of Plant Growth Regulators on Callus Proliferation

**Figure 4:**
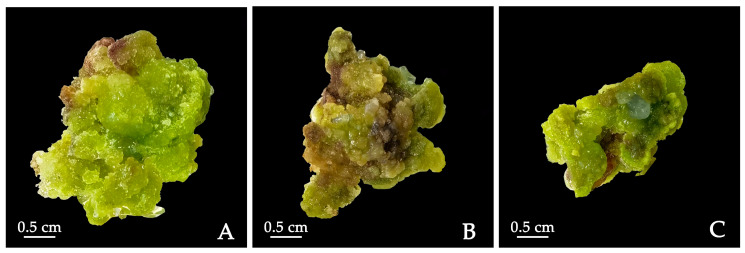
The proliferation status of calluses from cotyledon of *P. lactiflora* in different culture media. (**A**) In ½ MS + 1.0 mg·L^−1^ NAA + 1.0 mg·L^−1^ 2,4-D + 0.5 mg·L^−1^ TDZ medium. (**B**) In ½ MS + 0.5 mg·L^−1^ NAA + 0.1 mg·L^−1^ 2,4-D + 0.5 mg·L^−1^ TDZ medium. (**C**) In ½ MS + 0.1 mg·L^−1^ NAA + 0.5 mg·L^−1^ 2,4-D + 0.5 mg·L^−1^ TDZ medium. These results were obtained after 30 days of cultivation. 2,4-D: 2,4-Dichlorophenoxyacetic acid; NAA: 1-naphthaleneacetic acid; TDZ: thidiazuron.

It is well known that plant growth regulators are required to effectively promote callus proliferation. The callus induced in MS + 0.5 mg·L^−1^ TDZ + 0.5 mg·L^−1^ 2,4-D + 0.5 mg·L^−1^ NAA was selected as the material. We tested the impacts of a combination of two auxins (NAA and 2,4-D) with 0.5 mg·L^−1^ TDZ. Overall, the callus in the medium with NAA, 2,4-D and TDZ was vibrantly green with full and loose tissue after 20 days of growth (Figure 4A–C). Our results further show that the medium ½ MS + 1.0 mg·L^−1^ NAA + 1.0 mg·L^−1^ 2,4-D + 0.5 mg·L^−1^ TDZ had the highest proliferation coefficient of 3.13, followed by ½ MS + 0.5 mg·L^−1^ NAA + 0.1 mg·L^−1^ 2,4-D + 0.5 mg·L^−1^ TDZ, with a proliferation coefficient of 2.20 (Table 5). However, different degrees of browning occurred in different media, and the browning rate ranged from 4.33% to 58.33%. Taking both parameters into account, the best medium for callus proliferation was ½ MS + 1.0 mg·L^−1^ NAA + 1.0 mg·L^−1^ 2,4-D + 0.5 mg·L^−1^ TDZ.

### 2.6. Effects of Cytokinin Type and Concentration on Shoot Bud Differentiation

**Figure 5:**
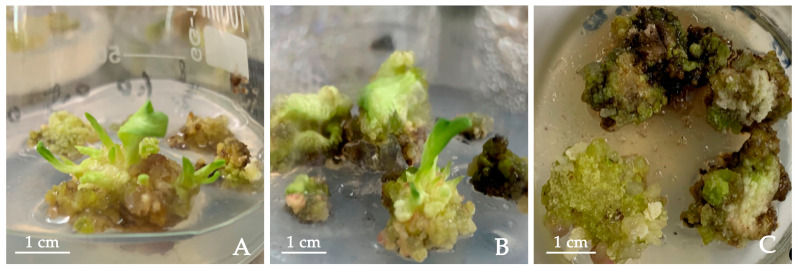
Shoot bud regeneration from calluses derived from cotyledon of *P. lactiflora* on MS + 0.2 mg·L^−1^ NAA + 1.0 mg·L^−1^ PVP with different cytokinins: (**A**) 0.5 mg·L^−1^ TDZ, (**B**) 0.5 mg·L^−1^ KT, (**C**) 0.5 mg·L^−1^ 6-BA. TDZ: thidiazuron; KT: kinetin; 6-BA: 6-Benzyladenine.

Cytokinins play an important role in shoot bud differentiation in tissue culturing. We tested the ability of the three cytokinins, i.e., TDZ, KT and 6-BA, to induce shoot bud differentiation and their effects on callus growth status in MS medium supplemented with 0.2 mg/L NAA and 1 mg/L PVP. First, adventitious buds were successfully generated and elongated in the medium containing 0.5 mg·L^−1^ TDZ with a 10.71% differentiation rate. The differentiation rate was lower in the medium containing 0.5 mg·L^−1^ KT (5.71%). A greater browning degree was observed for calluses cultured with high concentrations of BA. Little or moderate browning was observed when TDZ or KT was applied at the tested concentrations (Table 6). Second, the shoot bud differentiation state was the best in the medium containing 0.5 mg·L^−1^ TDZ, and a large number of green protrusions were generated on the surface of the callus. On average, each callus with a differentiation ability could generate 4.2 adventitious buds (Figure 5A). Adding 0.5 mg·L^−1^ KT did induce some calluses with a few protrusions on the surface that subsequently differentiated into new buds, and calluses with a differentiation ability could generate 2.3 adventitious buds/per callus. A total of 56 adventitious buds were induced on the medium supplemented with 0.5 mg·L^−1^ TDZ and 0.5 mg·L^−1^ KT. The conditions of the calluses worsened, developing a hard and compact texture (Figure 5B). Lastly, the medium containing 6-BA turned out to be undesirable for *P. lactiflora* tissue culturing, with the highest browning rate, worst callus state and no obvious differentiation (Figure 5C). In conclusion, MS + 0.5 mg·L^−1^ TDZ + 0.2 mg·L^−1^ NAA + 1.0 mg·L^−1^ PVP had the best performance in inducing shoot bud differentiation.

### 2.7. Effects of Auxin: Cytokinin Ratio on Shoot Development

One of the major difficulties faced by *P. lactiflora* is shoot bud regeneration and further proliferation of the adventitious buds, so it is critical to select the appropriate plant growth regulators for the growth of adventitious buds. Our testing showed that the addition of NAA and 6-BA in the medium could induce the growth of adventitious buds. More importantly, the ratio of NAA to 6-BA concentration greatly affected the growth state of adventitious buds. The smaller the NAA:6-BA ratio was, the better the growth potential of adventitious buds. We demonstrated that the MS medium containing 1.0 mg·L^−1^ GA_3_ + 0.5 mg·L^−1^ NAA + 1.0 mg·L^−1^ 6-BA led to the best stem pumping rate of adventitious buds (58.3%) and the highest stem height (2.54 cm), as well as the best leaf expansion rate (62.9%) (Table 7). The effect of this medium on the growth of adventitious buds was significantly better than that of the other two media.

### 2.8. Effects of Auxin Type and Concentration on Rooting Induction

**Figure 6:**
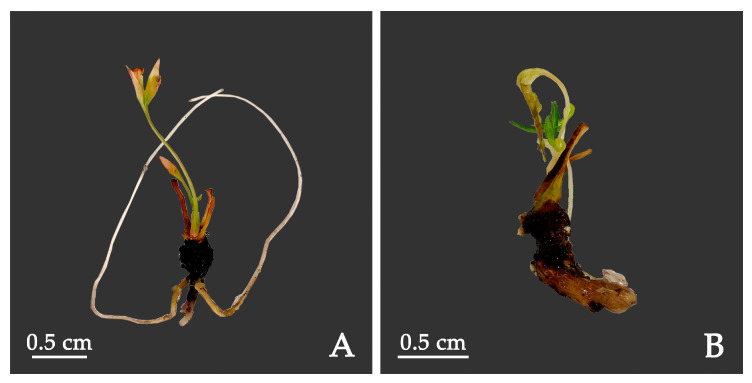
*P. lactiflora* shoots rooted in ½ MS + 3 g/L AC medium with different auxins: (**A**) 1.0 mg·L^−1^ IBA, (**B**) 0.5 mg·L^−1^ IAA+ 0.5 mg·L^−1^ IBA. The images shown are of plantlets after 45 days in rooting medium. IAA: indole-3-acetic acid; IBA: indole-3-butyric acid.

Under tissue culture conditions, auxins are used to induce root development. We explored the effects of auxin types and their concentrations on inducing rooting in *P. lactiflora* flora in a medium composed of ½ MS + 3.0 g·L^−1^ AC. No rooting (0%) was obtained when no auxins were added to the medium. Our test indicate that after 10 days of shoot inoculation, the adventitious roots began to elongate, but the roots were weak. After 20 days, the roots grew thicker and some explants had multiple roots. IAA and IBA had different effects on the induction of adventitious roots. With the same concentration, the ability of IBA to induce roots was stronger than that of IAA. When the concentration of IBA was 1.0 mg·L^−1^, the induction rate of roots was the highest (38.89%) (Table 8). The adventitious roots in this medium were in the best state, and the number and the coverage area of roots were large (Figure 6A). When 0.5 mg·L^−1^ IAA and 0.5 mg·L^−1^ IBA were added, rooting was difficult, and the roots were short (Figure 6B). When the IAA concentration was the same, the higher IBA concentration led to a higher rooting rate. When the concentrations of IAA and IBA were 1.0 mg·L^−1^, the rooting rate reached 31.11%.

## 3. Discussion

The success of callus induction in *P. lactiflora* is related to the explant type, culture conditions, basic media and plant growth regulators. There are great differences in the states of calluses formed from different explants [31]. Studies have shown that cotyledons under suitable conditions can efficiently form high-quality calluses [24,32]. Coincidently, we discovered that using cotyledons as explants resulted in a higher rate of callus induction and a lower rate of browning. We observed that there was no significant difference in induction rate when callus were treated under dark or light conditions. However, it has been documented that *Rosa hybrida*, lotus and *Datura innoxia* have high callus induction rates only in dark environments [33,34,35]. Our findings indicate that the explants not subjected to dark culturing were not completely dedifferentiated, and dark culturing for 20 days was more suitable for callus growth. This observation aligns with previous studies on *Rosa hybrida* and *Clivia miniata*, which also reported high callus quality under dark conditions [33,36]. We demonstrated that the MS medium significantly increased the induction speed of calluses, reduced the browning rate, and guaranteed a higher callus quality. This is similar to the results of studies on *Moso Bamboo*, *Toona ciliata* and *Ethiopian mustard* [32,37,38]. Furthermore, our experiment has revealed that the concentration of NAA influenced both the rate of callus induction and the time of callus emergence. This observation is consistent with the findings of Guo et al. (2021), who reported a positive effect of NAA on callus induction in *Abies koreana* [39]. However, it is worth noting that the previous study did not find a significant effect of NAA concentration on the formation time of black cumin callus [40]. Many studies have found that PIC plays an important role in the induction of callus [41,42,43,44]. We consistently observed that the addition of 4 mg·L^−1^ PIC into the medium induces more callus, with light yellow particles on the surface, which is similar to the results for peony [45]. However, when the PIC concentration was higher, the browning rate of the callus was also increased. Therefore, we should be sure that the appropriate concentration of PIC is applied to reduce the browning rate of callus.

The callus proliferation of *P. lactiflora* is primarily influenced by plant growth regulators. Our study has revealed that the combination of 1.0 mg·L^−1^ NAA, 1.0 mg·L^−1^ 2,4-D and 0.5 mg·L^−1^ TDZ increased the callus proliferation coefficient of *P. lactiflora* to 3.13, and reduced the browning rate to 10.33%. Previous research has also demonstrated that the combination of NAA, 2,4-D and TDZ is beneficial to the callus proliferation of peony and *Dendrocalamus luodianense* [46,47]. Interestingly, the combination of NAA and TDZ led to the highest callus proliferation rate of *Clivia miniata* [36]. In our experiment, the appropriate concentration of plant growth regulators significantly reduced the degree of browning during callus proliferation. Lower browning rates were observed when the concentration ratio of 2,4-D to NAA was 1:1 or 2:1. At the same time, we found that the frequent transferring of callus can also reduce callus browning.

The formation of adventitious shoot buds from callus of *P. lactiflora* is a challenging process primarily influenced by plant growth regulators. Both cytokinin and auxin can promote the formation and proliferation of shoot buds [48,49,50,51,52]. Our research demonstrated that TDZ combined with NAA successfully achieved the differentiation of adventitious shoot buds, and the buds grew robustly. These findings align with previous studies on the differentiation of adventitious buds in *Pogostemon cablin*, *Cotoneaster wilsonii* and *Allium hirtifolium* [53,54,55]. In the study of *Cotoneaster wilsonii*, the effect of TDZ on adventitious bud differentiation was better than that of 6-BA [54]. Our experiments have also confirmed this finding. When the concentration of TDZ was 0.5 mg·L^−1^, the shoot buds differentiation rate of *P. lactiflora* was the best. When the concentration of TDZ increased to 1.0 mg·L^−1^, the differentiation of adventitious buds of *P. lactiflora* was very difficult, although it was found that a small amount of callus of *P. lactiflora* could differentiate into adventitious buds when 1.0 mg·L^−1^ TDZ was added [24,25]. The main reason for the low differentiation rate of adventitious buds of *P. lactiflora* is not only related to the combination and concentration of plant growth regulators, but also may be related to the number of callus subcultures. Multiple subculturing results in a reduction in meristematic activity, impeding the differentiation of calluses into organ primordia.

IBA, NAA and IAA are commonly used as plant growth regulators for rooting in plant tissue culture systems [56,57,58]. Our results show that the addition of IAA or IBA to ½ MS media could significantly increase the rooting rate of *P. lactiflora*, which is consistent with the findings of the study of Zhao et al. [59]. Nevertheless, a prior investigation revealed that ½ MS media without plant growth regulators could also promote the root development of seedlings in *P. lactiflora* [9]. The rooting rate of peony was the highest in the combination of IBA and IAA, and was better than that in IAA or IBA alone [60]. Interestingly, we observed the opposite results, and found that, compared with the combination of IAA and IBA, the rooting induction rate of *P. lactiflora* with IBA alone was the highest. Overall, IBA treatment had the best rooting effect for *P. lactiflora*, which is similar to the results derived for *Petunia hybrida* and *Oryza sativa* [61,62].

The establishment of a *P. lactiflora* regeneration system has long been challenging. Although we have made some progress on the regeneration of *P. lactiflora*, the rates of adventitious bud differentiation and rooting in *P. lactiflora* are still relatively low. Thus, the efficient establishment of a *P. lactiflora* regeneration system still has a long way to go, and further exploration is necessary to effectively achieve the regeneration of *P. lactiflora* via the callus pathway.

## 4. Materials and Methods

### 4.1. Plant Material and Culture Conditions

The intervarietal hybrid seeds “Fen Yunu” ♀ × “Fen Yulou” ♂ were used as materials, and were collected via pollination from the germplasm resources nursery in Shenyang Agricultural University, Liaoning province, China. The seeds that were collected 100 days after pollination in the current year were shade-dried, and stored in a refrigerator at 4 °C. *P. lactiflora* hybrid seeds were soaked in water for 48 h, and we then peeled off the seed coats. Experiments of embryo germination were performed according to the protocol of Duan et al. [22] with some modifications. Subsequently, seeds were treated with 75% ethanol for 30 s and sterilized with 0.1% (*w*/*v*) HgCl_2_ for 5–6 min followed by washing 4 times with sterile distilled water. Mature zygotic embryos were cut from seeds and inoculated in MS medium supplemented with 0.5 mg·L^−1^ GA_3_ and 1.0 mg·L^−1^ 6-BA. After 20 days of culturing, the produced hypocotyls and cotyledons were used as explants for subsequent experiments. All culture media were added with 30 g/L sucrose and 6 g/L agar with pH of 5.8 and autoclaved at 121 °C for 20 min. Except for those subjected to special instructions, all cultures were maintained at 25 ± 1 °C, in a 16/8 h (light/dark) photoperiod with 2000–3000 lx light intensity. All components of the culture media used in this article were purchased from Beijing Solarbio Science & Technology Co., LTD (Beijing, China). All plant growth regulators (PGRs) were purchased from Shanghai Yien Chemical Technology Co., LTD (Shanghai, China).

### 4.2. Callus Induction by Explant Types and Auxins Combined with 6-BA

The zygotic embryos, cotyledons, and hypocotyls were cultured in MS medium supplemented with 1.0 mg·L^−1^ BA combined with 2.0 mg·L^−1^ PIC, 1.0 mg·L^−1^ BA combined with 2.0 mg·L^−1^ 2,4-D or 1.0 mg·L^−1^ BA combined with 1.0 mg·L^−1^ NAA, respectively, to induce calluses under dark conditions. After 45 days, the callus induction rates of the three explants on different media were observed.

### 4.3. Callus Induction by Basal Medium and NAA Concentration

Experiments on callus induction were performed according to the protocol of Duan et al. [22] with some modifications. The cotyledons were cultured in MS and ½ MS medium supplemented with 0.5 mg·L^−1^ TDZ, 0.5 mg·L^−1^ 2,4-D and NAA (0.1, 0.3, 0.5, 1.0 mg·L^−1^), respectively. After 30 days, the callus induction rate and browning rate were calculated, and the growth status of the callus in each medium was observed.

### 4.4. Callus Induction by Dark Culture Time

The cotyledons were transferred to MS medium supplemented with 0.5 mg·L^−1^ TDZ, 0.5 mg·L^−1^ 2,4-D and 0.5 mg·L^−1^ NAA and cultured in the dark for 10, 20 and 30 days, respectively, while normal light culturing was used as the control treatment (16 h light/8 h dark photoperiod with 2000–3000 lx light intensity). After dark culturing, they were transferred to light culturing conditions. After 30 days, the callus induction of cotyledons under different dark culturing times was observed.

### 4.5. Callus Induction by PIC Concentration

Cotyledons were cultured in ½ MS medium supplemented with 1.0 mg·L^−1^ TDZ + 1.0 mg·L^−1^ 2,4-D + 0.5 g·L^−1^ CH + 1.0 mg·L^−1^ PVP for 30 days to produce calluses, and then transferred to ½ MS medium supplemented with 1.0 mg·L^−1^ TDZ + 1.0 mg·L^−1^ NAA + 0.5 g·L^−1^ CH + 1.0 mg·L^−1^ PVP for callus proliferation. After 30 days, the obtained calluses were transferred to ½ MS (Ca^2+^) medium supplemented with 1.0 mg·L^−1^ TDZ + 0.5 g·L^−1^ CH + 1.0 mg·L^−1^ PVP + PIC (0, 2.0, 4.0 mg·L^−1^), and the calluses were induced under dark conditions. After 30 days, the induction of calluses was observed.

### 4.6. Callus Proliferation

The calluses induced in MS + 0.5 mg·L^−1^ TDZ + 0.5 mg·L^−1^ 2,4-D + 0.5 mg·L^−1^ NAA were selected as the material. Experiments on callus proliferation were performed according to the protocol of Duan et al. [22] with some modifications. The most well-grown calluses were cut into 1 cm squares and transferred to the ½ MS medium supplemented with different concentrations of NAA (0.1, 0.5, 1.0 mg·L^−1^), 2,4-D (0.1, 0.5, 1.0 mg·L^−1^) and TDZ (0.5 mg·L^−1^) for callus proliferation. Each treatment had 20 explants and 3 replicates. After 30 days, the callus proliferation coefficient and browning rate were measured.

### 4.7. Adventitious Bud Induction

The callus proliferated in ½ MS + 1.0 mg·L^−1^ NAA + 1.0 mg·L^−1^ 2,4-D + 0.5 mg·L^−1^ TDZ was used as the material. Experiments on adventitious bud induction were performed according to the protocol of Sun et al. [24] with some modifications. Calluses were transferred to MS medium containing 0.2 mg·L^−1^ NAA combined with TDZ (0.1, 0.3, 0.5, 1.0 mg·L^−1^), 0.2 mg·L^−1^ NAA combined with 6-BA (0.1, 0.3, 0.5, 1.0 mg·L^−1^) or 0.2 mg·L^−1^ NAA combined with KT (0.1, 0.3, 0.5, 1.0 mg·L^−1^) for shoot bud differentiation. The medium was supplemented with 1.0 mg·L^−1^ PVP to prevent browning. After 45 days, the shoot bud differentiation in each medium was observed.

### 4.8. Adventitious Bud Growth

Experiments on adventitious bud growth were performed according to the protocol of Liu et al. [63] with some modifications. The adventitious buds were transferred into MS medium with 1.0 mg·L^−1^ GA_3_ and tested at different concentrations and ratios of NAA and BA (1:1, 0.5:1 and 1:0.5, in mg·L^−1^). Each treatment had 20 explants and 3 replicates. After 30 days, the stem pumping rate, leaf expansion rate and shoot height of adventitious buds were recorded.

### 4.9. Root Induction

The shoots with 2–4 leaves and which were 1.5–2.5 cm in height were selected as materials. The shoots cultured for 30 d were transferred to ½ MS medium supplemented with 3.0 g·L^−1^ AC combined with different concentrations of IAA (0, 0.5, 1.0 mg·L^−1^) and IBA (0, 0.5, 1.0 mg·L^−1^) for root induction. The rooting was observed after 45 days.

### 4.10. Statistical Analysis

Except as noted, each treatment had 30 explants and 3 replicates. Statistical significance was calculated using SPSS software (Version 26.0) with the one-way analysis of variance (ANOVA) method, and significant difference was defined by Duncan’s multiple range test at *p* < 0.05. The significant data involved in this paper were calculated as follows: induction rate = (number of induced explants/total number of explants) × 100%; proliferation coefficient = fresh proliferation callus weight/inoculated callus weight; browning rate = (number of browning explants/total number of explants) × 100%; differentiation rate = (number of differentiated explants/total number of explants) × 100%; stem pumping rate: (number of stem pumping explants/total number of explants) × 100%; leaf expansion rate: (number of stems with expanded leaves/total number of stems) × 100%; rooting rate = (number of rooting explants/total number of explants) × 100%.

## 5. Conclusions

In summary, our study established an efficient callus induction method by manipulating plant growth regulators. We developed a method that allows us to obtain and proliferate calluses from cotyledons of *P. lactiflora*, and that resulted in indirect adventitious bud regeneration and their elongation to obtain shoots that were rooted, allowing us to obtain complete plants. The results of our system provide a new method for tissue culturing and the subsequent regeneration of *P. lactiflora*. However, the bud differentiation rate and rooting rate were low, which is a potential research avenue to be addressed in the future.

## Figures and Tables

**Table 1:** Effects of explant types and auxins combined with 6-BA on callus induction of *P. lactiflora*.

Explant Types	Plant Growth Regulators	Explants with Callus (%)
Cotyledon	1.0 mg·L^−1^ 6-BA + 2.0 mg·L^−1^ PIC	53.12 ± 5.64 b
	1.0 mg·L^−1^ 6-BA + 2.0 mg·L^−1^ 2,4-D	98.89 ± 1.92 a
	1.0 mg·L^−1^ 6-BA + 1.0 mg·L^−1^ NAA	90.26 ± 4.44 a
Hypocotyl	1.0 mg·L^−1^ 6-BA + 2.0 mg·L^−1^ PIC	26.67 ± 5.77 b
	1.0 mg·L^−1^ 6-BA + 2.0 mg·L^−1^ 2,4-D	85.56 ± 0.96 a
	1.0 mg·L^−1^ 6-BA + 1.0 mg·L^−1^ NAA	83.89 ± 3.47 a
Embryo	1.0 mg·L^−1^ 6-BA + 2.0 mg·L^−1^ PIC	29.44 ± 4.19 b
	1.0 mg·L^−1^ 6-BA + 2.0 mg·L^−1^ 2,4-D	85.56 ± 6.48 a
	1.0 mg·L^−1^ 6-BA + 1.0 mg·L^−1^ NAA	89.44 ± 4.19 a

The values represent mean ± SD. Different letters represent significant differences in the callus induction rate under different auxin conditions in the same explant, according to Duncan’s multiple range test at *p* < 0.05. The data presented in the table were obtained after 45 days of cultivation. 6-BA: 6-benzyleaminopurine; PIC: picloram; 2,4-D: 2,4-Dichlorophenoxyacetic acid; NAA: 1-naphthaleneacetic acid.

**Table 2:** Effects of basal media and NAA concentration on callus induction from cotyledon of *P. lactiflora*.

Culture Media	Plant Growth Regulators (mg·L^−1^)			Callus Induction Time (d)	Induction Percentage (%)	Browning Percentage (%)	Callus Induction State
	TDZ	2,4-D	NAA				
MS	0.5	0.5	0.1	8–10	90.56 ± 4.19 b	8.89 ± 5.36 a	Light yellowish-green, compact, hard
			0.3	5–7	93.89 ± 3.47 ab	4.44 ± 5.09 a	Yellowish-green, compact, moderately hard
			0.5	3–5	98.33 ± 2.89 a	1.67 ± 2.89 a	Yellowish-green, compact, moderately hard
			1.0	3–5	97.22 ± 2.55 a	1.11 ± 1.92 a	Light emerald-green, flocculent, soft
½ MS	0.5	0.5	0.1	10–15	79.44 ± 4.19 b	11.11 ± 5.09 b	Green, compact, hard
			0.3	10–15	82.22 ± 6.31 b	10.00 ± 5.00 b	Yellowish-green, compact, moderately hard
			0.5	5–10	91.67 ± 2.89 a	21.11 ± 5.35 a	Light yellowish-green, compact, brown protrusions
			1.0	5–8	93.33 ± 5.77 a	10.00 ± 3.33 b	Yellowish-green, compact, moderately hard

The values represent mean ± SD. Different letters represent significant differences in callus induction rate and browning rate under different concentrations of NAA in the same medium, according to Duncan’s multiple range test at *p* < 0.05. The data shown were obtained after 30 days of cultivation. MS: Murashige and Skoog; TDZ: thidiazuron; 2,4-D: 2,4-Dichlorophenoxyacetic acid; NAA: 1-naphthaleneacetic acid.

**Table 3:** Effects of dark culturing time on callus induction from cotyledon of *P. lactiflora*.

Dark Culture Time (d)	Induction Percentage (%)	Browning Percentage (%)
0	90.44 ± 6.48 a	8.89 ± 5.36 c
10	88.67 ± 8.08 a	24.89 ± 4.29 b
20	91.33 ± 4.16 a	15.67 ± 4.04 c
30	87.33 ± 6.43 a	40.56 ± 4.19 a

The values represent mean ± SD. Different letters mean significant differences according to Duncan’s multiple range test at *p* < 0.05. The data presented in the table were obtained after 30 days of cultivation.

**Table 4:** Effects of PIC on callus induction from cotyledon of *P. lactiflora*.

PIC Concentration (mg·L^−1^)	Induction Percentage (%)	Browning Percentage (%)
0	20.00 ± 5.00 b	15.00 ± 5.00 c
2	46.67 ± 2.89 a	33.33 ± 2.89 b
4	48.33 ± 5.77 a	46.67 ± 7.63 a

The values represent mean ± SD. Different letters mean significant differences according to Duncan’s multiple range test at *p* < 0.05. The data presented in the table were obtained after 30 days of cultivation. PIC: picloram.

**Table 5:** Effects of NAA and 2,4-D on *P. lactiflora* callus proliferation and browning.

Concentration (mg·L^−1^)			Callus Proliferation Coefficient	Browning Percentage (%)
2,4-D	NAA	TDZ		
0.1	0.1	0.5	1.87 ± 0.11 bc	10.33 ± 1.53 e
0.1	0.5	0.5	2.20 ± 0.29 b	20.67 ± 3.06 d
0.1	1.0	0.5	1.80 ± 0.02 cd	30.33 ± 2.52 c
0.5	0.1	0.5	1.59 ± 0.21 d	58.33 ± 3.79 a
0.5	0.5	0.5	1.83 ± 0.12 cd	4.33 ± 2.08 e
0.5	1.0	0.5	1.92 ± 0.19 bc	20.67 ± 4.04 d
1.0	0.1	0.5	1.83 ± 0.16 cd	39.33 ± 3.06 b
1.0	0.5	0.5	2.10 ± 0.19 bc	9.67 ± 2.52 e
1.0	1.0	0.5	3.13 ± 0.13 a	10.33 ± 5.51 e

The values represent mean ± SD. Different letters mean significant differences according to Duncan’s multiple range test at *p* < 0.05. The data presented in the table were obtained after 30 days of cultivation. 2,4-D: 2,4-Dichlorophenoxyacetic acid; NAA: 1-naphthaleneacetic acid; TDZ: thidiazuron.

**Table 6:** Types and concentrations of cytokinins and their effects on callus growth and indirect shoot bud differentiation.

Type and Concentration of Cytokinins		Browning Degree	Shoot Bud Differentiation Percentage (%)
TDZ	0.1	slight browning	0.00 ± 0.00 c
	0.3	slight browning	0.00 ± 0.00 c
	0.5	slight browning	10.71 ± 2.34 a
	1.0	moderate browning	0.00 ± 0.00 c
6-BA	0.1	slight browning	0.00 ± 0.00 c
	0.3	moderate browning	0.00 ± 0.00 c
	0.5	extreme browning	0.00 ± 0.00 c
	1.0	extreme browning	0.00 ± 0.00 c
KT	0.1	slight browning	0.00 ± 0.00 c
	0.3	slight browning	0.00 ± 0.00 c
	0.5	slight browning	5.71 ± 2.07 b
	1.0	moderate browning	0.00 ± 0.00 c

The values represent mean ± SD. Different letters mean significant differences according to Duncan’s multiple range test at *p* < 0.05. In this experiment the MS + 0.2 mg/L NAA + 1 mg/L PVP culture medium was used. TDZ: thidiazuron, 6-BA: 6-Benzyladenine; KT: kinetin.

**Table 7:** Effects of auxin:cytokinin ratio on adventitious bud growth from calluses derived from cotyledons of *P. lactiflora*.

Plant Growth Regulators	Stem Pumping Percentage (%)	Leaf Expansion Percentage (%)	Stem Height (cm)
1.0 mg·L^−1^ NAA: 1.0 mg·L^−1^ 6-BA	35.0 ± 5.00 b	71.4 ± 3.14 a	1.74 ± 0.18 b
0.5 mg·L^−1^ NAA: 1.0 mg·L^−1^ 6-BA	58.3 ± 2.89 a	62.9 ± 2.59 b	2.54 ± 0.19 a
1.0 mg·L^−1^ NAA: 0.5 mg·L^−1^ 6-BA	30.0 ± 8.66 b	44.4 ± 4.13 c	1.58 ± 0.13 b

The values represent mean ± SD. Different letters indicate significant differences according to Duncan’s multiple range test at *p* < 0.05. These results were obtained after 30 days. NAA: 1-naphthaleneacetic acid; 6-BA: 6-benzyleaminopurine.

**Table 8:** Effects of IAA and IBA on rooting induction from shoots of *P. lactiflora*.

Concentration (mg·L^−1^)		Rooting Percentage (%)	Average Root Number	Longest Root Length (cm)
IAA	IBA			
0	0	0.00 ± 0.00 e	0.00 ± 0.00 d	0.00 ± 0.00 e
0.5	0.5	7.78 ± 1.92 e	1.39 ± 0.10 c	1.55 ± 0.12 d
0.5	1.0	26.67 ± 3.34 bc	2.29 ± 0.04 b	2.02 ± 0.10 c
1.0	0.5	22.22 ± 3.85 cd	2.14 ± 0.13 b	2.04 ± 0.06 c
1.0	1.0	31.11 ± 6.94 ab	2.30 ± 0.14 b	3.05 ± 0.08 b
0	1.0	38.89 ± 5.09 a	3.13 ± 0.17 a	3.40 ± 0.11 a
1.0	0	17.78 ± 1.92 d	1.62 ± 0.20 c	1.92 ± 0.26 c

The values represent mean ± SD. Different letters indicate significant differences according to Duncan’s multiple range test at *p* < 0.05. In this experiment the ½ MS + 3.0 g/L AC medium was used, and these results were obtained after 45 days of cultivation. IAA: indole-3-acetic acid; IBA: indole-3-butyric acid.

## Data Availability

The data presented in this study are available in this article.
